# Structural Factors Responsible for Universal Health Coverage in Low- and Middle-Income Countries: Results From 118 Countries

**DOI:** 10.3389/fpubh.2019.00414

**Published:** 2020-01-21

**Authors:** Chhabi Lal Ranabhat, Mihajlo Jakovljevic, Meghnath Dhimal, Chun-Bae Kim

**Affiliations:** ^1^Policy Research Institute, Kathmandu, Nepal; ^2^Department of Public Health, Manmohan Memorial Institute of Health Science, Kathmandu, Nepal; ^3^Department of Global Health Economics and Policy, University of Kragujevac, Kragujevac, Serbia; ^4^Division of Health Economics, Lund University, Lund, Sweden; ^5^Department of Public Health and Healthcare, First Moscow State Medical University (Sechenov University), Moscow, Russia; ^6^Nepal Health Research Council, Ramshahpath, Kathmandu, Nepal; ^7^Department of Preventive Medicine, Yonsei University Wonju College of Medicine, Wonju, South Korea; ^8^Hongcheon County Hypertension and Diabetes Registration and Education Center, Hongcheon, South Korea; ^9^Institute for Poverty Alleviation and International Development, Yonsei University, Wonju, South Korea

**Keywords:** economy, sociodemographic factors, governance, political stability, low- and middle-income countries, universal health coverage

## Abstract

**Background:** Demography, politics, economy, and governance appear to be the major structural factors for health and well-being. These factors have a significant role to play in achieving universal health coverage (UHC). The majority of previous studies did not highlight those factors. The aim of this study is to explore the basic structural factors (political stability, demography, gross national income, governance, and transparency) associated with a UHC index of low- and middle-income countries because for a long time there has be a stagnation achieving universal health coverage.

**Methodology:** This study was a cross-sectional study applying multiple indices as variables. Low- and middle-income countries' selected indicators were the study variables. Data concerned the current political stability, sociodemographic status, gross national income (GNI), and governance status as independent variables and the UHC index of the countries as the dependent variable. Mean and standard deviations were used for the average values of the variables, a raw correlation was shown among variables and a hierarchical linear regression model was used for multi variate analysis.

**Results:** Government health expenditure is 6% out of the total budget in upper middle countries (UMIC) and ~5% in lower middle countries (LMIC) and low-income countries (LIC), population below poverty line is more than 2-fold higher in LIC in comparison with high income countries, UHC index, and socio-demographic index (SDI) index is similar in LMIC and LIC and slightly higher in UMIC. There is a positive association between government health expenditure, governance index, stability index, the SDI index, and GNI per capita and a negative association between populations below poverty line with UHC index. According to our full regression analysis model, governance, stability, and SDI index were associated with a significantly increased UHC index by 0.33, 0.41, and 0.57 (*p* < 0.05).

**Conclusion:** To achieve UHC, good governance, political stability, and demographic balance are prerequisites and addressing these factors would help to meet by 2030 across countries.

## Introduction

Universal health coverage (UHC) is about ensuring that people have access to the health care they need without suffering financial hardship. Fundamentally it is related to the contribution and distribution of resources to people with justice. Health is a foundational investment in human capital and in economic growth—without good health, children are unable to go to school and adults are unable to go to work. In other words, financing on health care is not an expenditure, it is an investment for human capital. In low- and middle-income countries, there are some debates about investing in health care and misunderstandings that illness occurs due to individual careless and they have to solve the problem themselves (1). It is a stereotype of thinking in the implementation of universal health coverage. This understanding creates obstacles, social and political instability (2), poor governance (3), and lack of transparency (4). Those situations create significant challenges to quality of health service, population, and financial coverage which are the three pillar of universal health coverage.

### Situation of Low- and Middle-Income Countries and Health

Epidemiology of low- and middle-income countries shows that the economic and magnitude of the disease burden is increasing. They have been fighting with communicable diseases for many years e.g., in Africa many infectious diseases have emerged or reemerged in the twenty-first century (5), they are now stuck with non-communicable diseases and injuries (6) and reproductive health problems resulting in a triple burden. Moreover, LMIC are suffering from improper priority setting, strategic plans, and cost minimization approach to enhance sustainability of the health care system (7). According to the estimates of the World Health Organization, by 2020, non-communicable diseases like diabetes mellitus, cancer, obesity, chronic obstructive pulmonary disease (COPD) etc. will account for 80% of the global burden of disease and 70% of deaths- the majority of the burden and death being in low and middle-income countries (8, 9). The increasing urbanization trend and life style factors, like sedentary life style, practice of fast, and junk food increase the burden of non-communicable disease in LMIC (10). Likewise, demography of LMIC is unpredictable because there is high international migration, the middle income population is increasing (11), and the elder population is also increasing (12). The economy of LMIC is in a stagnation situation because the gross national product is low, high income countries occupied their business all over the world and there is difficulty in competing with high income countries (13). The politics of LMIC is mostly unstable due to fluctuation tendency of the middle-income group, dynasty in politics, and insufficient democratic practice. There is uncertainty for middle income group people. They are sometimes aligned with the rich group and sometimes with marginalized people and there is always the possibility for uncertainty to the form of political alliance and power (14). The dynasty politics creates a deep root power structure and it is very difficult to manage and creates instability (15). Moreover, there are some unethical practice in elections and there is no fairness and there is always a possibility for instability and large number of people get confused and divided (16). So, the mindset of the majority of the people is to always feel insufficiency and instability (17). Due to high out of pocket payments, poor achievement of prepaid health insurance, high cost of health care, it is difficult to achieve UHC in LMIC. Even for government, there is an equal challenge to increase budgets for health and quality health service and maintain equity in health care.

Previous studies focused mostly on core and technical aspects regarding universal health coverage. Usually, the variables analyzed have been economic and wealth status with universal coverage. Regression is performed and the conclusion is significant positively association. Researchers have few concerns about the how of sustainable economic growth. Studies have shown that the economy would be sustainable after the guarantee of health service to all people (18, 19). Poor enrollment and drop out in health insurance would significantly affect universal health coverage (20). Likewise, increasing government health expenditures would contribute to quality health service and also coverage (18, 21, 22). Out of pocket expenditure increase catastrophic health expenditure and directly push into poverty (23, 24). Poor people need more health service, but they are not able to pay. On the other hand, there is wide variation in the cost of health care by different health care facilities and people cannot decide receive health care where health care facilities are affordable, accessible, and provide quality health service (25). In this situation, a confusion creates, and people are susceptible for out-of-pocket expenditure. Most of the researcher highlighted the above aspects regarding universal health coverage locally, nationally, and globally.

### Philosophical and Structural Factors Toward Health Care

In low- and middle-income countries, obviously, there are not enough resources and a need to create some sort approach to reduce the lack in each sector (26). The result is always a situation of individualistic mind set. To develop and form a mindset there are different structural factors like belief system, culture, regular network, and communication, geographical status, update with global phenomenon etc. (27) In all situations, individualistic mind set may not be the problem but not always. Two Japanese scholars concluded in their studies that individualistic mind set effects interpersonal relationships, feelings of insecurity, and creates many doubts in Asians but there is less impact for Americans (28). Likewise, Gustavsson concluded that individualism negatively effects civic virtues particularly for the power holders (29). As a result, power holders deviate from their role and responsibility and lose their efficient management capacity. A study on theory of mind by Vu et al. explored whether individualism creating feelings of insecurity ultimately shows unstable behavior and influences in society and group (30). Nordström et al. reveled that health is fundamentally a personal matter and each person must be ready to manage their self during illness (31). The results of this study indicate that individuals are solely responsibility for their own health and there are few roles for the environment and health care systems. Okely et al. concluded in his research that individualism is a great barrier to well-being (32) and it has multiple impacts on health education, social security, social capital etc.

**Figure 1:**
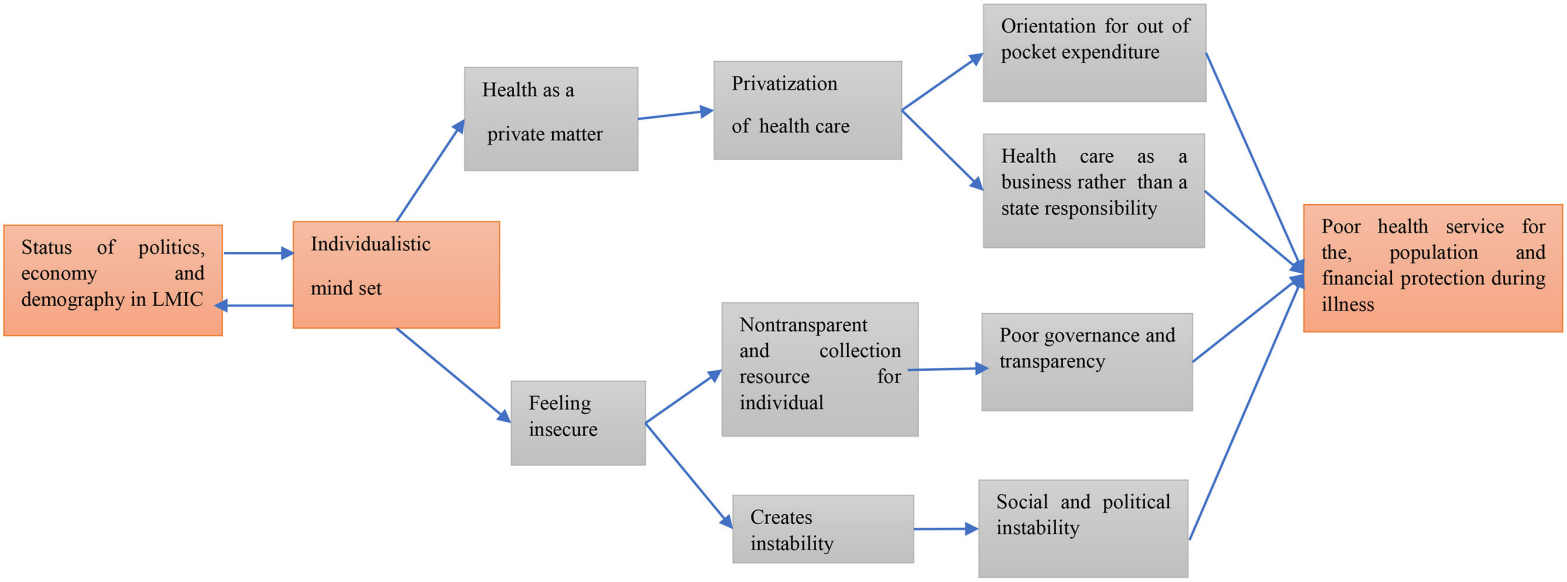
Structural phenomenon associated with universal health coverage (UHC).

The above literature emphasizes that on the one hand individual mind sets see health as a personal matter. Such a concept encourages privatization of health and it ultimately increases the out of pocket expenditure and promotes health care as a business. From this result, society becomes polarized and ultimately long-term barriers to universal health coverage are created. On the other hand, an individual mind set feels insecure at any time and instability forms in every mind and ultimately this creates conflict in society and a culture of non-transparent corruption and consumption of public resources by the private sector results in poor health delivery. These consequences, particularly in low- and middle-income countries result in a state with poor economy and social capital and achieving UHC is very far from reality (Figure 1).

It is apparent that under the WHO initiative the development of action plans across all countries including LMICs regarding strategies to reduce AMR, as well as morbidity/mortality due to non-communicable diseases like cardiovascular diseases, diabetes, and others. However, there is very little research regarding UHC analyzing structural factors related to UHC. The bulk of the analysis of health in the developing world has emphasize technical issues such as disease prevention and policy design, with comparatively little attention paid to the political and economic dynamics that influence the adoption, implementation, and on-going progress of health reforms. Basically, the structural factors are related to politics, economy, demographic situation, resource handling for health care, and understanding of universal health care. The aim of this study is to explore the basic structural factors (political stability, demography, gross national income, governance, and transparency) associated with the UHC index of low- and middle-income countries.

## Methodology

### Study Design

This study is a cross-sectional study with multiple indexes as variables. Mostly a few sample sized data sets have been examined through non-parametric tests and sometimes dissimilar types of data like Chen et al. (33). The method was data accumulation/assembly from different sources through internet tools. For this study data were used from different sources openly available on official web pages of some organization and collected for their own purpose.

### Study Unit

The analysis involves countries as the study unit and all study variables are the mean value of those countries.

### Number of Countries Involves in Research

There were 138 countries which have been categorized as low- and middle-income countries (lower income economies−31, lower middle countries−47, and upper-income countries−60) (34). But not all of these countries had data and 20 countries were excluded due to unavailability of data on the variables of interest. We included 118 low- and middle-income countries for the analysis.

### Data Sources

Data were received from different organizations and collected for their own purpose. Particularly, data on the transparency status as the independent variables and those variables were a measurement of structural factors of LMIC. For the dependent variable, the UHC index of those countries was used. The details on the variables are described below:

1. Political stability index: The index included political stability and absence of violence/terrorism measures, perceptions of the likelihood that the government will be destabilized or overthrown by unconstitutional or violent means, including politically motivated violence and terrorism. The index also includes an average of several other indexes from the Economist Intelligence Unit (EIU), the World Economic Forum (WEF), and the Political Risk Services (PRS), among others (35). The underlying indexes reflect the likelihood of a disorderly transfer of government power, armed conflict, violent demonstrations, social unrest, international tensions, terrorism, as well as ethnic, religious or regional conflicts. The methodology of the overall index is kept consistent, so the numbers are comparable over time. The data of LMIC by country was calculated from 2000 to 2015.
2. Gross national income (GNI) per capita PPP: It is gross national income (GNI) was converted to international dollars using purchasing power parity rates. An international dollar has the same purchasing power over GNI as a U.S. dollar has in the United States. GNI is the sum of value added by all resident producers plus any product taxes (less subsidies) not included in the valuation of output plus net receipts of primary income (compensation of employees and property income) from abroad (36). It should reflect the average before tax income of a country's citizens. A country's GNI per capita tends to be closely linked with other indicators that measure the social, economic, and environmental well-being of the country and its people. The estimated average of 2000–2015 of each country was used.
3. Governance index: The Governance Index is comprised of five criteria, which are based on a total of 20 indicators (37). It focuses on how effectively policymakers facilitate and steer development and transformation processes. By examining and evaluating decision-makers' reform policies, the BTI sheds light on those factors determining success and failure on the way to democracy and a market economy. Successful transformation management implies that governments are consistent in pursuing their goals and use their resources wisely and effectively. It also implies that decision-makers cultivate the broadest possible consensus for their transformation goals and work reliably with external supporters and neighboring states. The performance between 2015 and 2017 was used.
4. Government health expenditure (GHE): Government health expenditure is the sum of public health expenditure. It covers the provision of health services (preventive and curative), family planning activities, nutrition activities, and emergency aid designated for health but does not include provision of water and sanitation (38). The GHE of each country was the average means for 2000–2015 of each country.
5. Universal health coverage (UHC) index: The index was constructed from geometric means of the 14 tracer indicators (Family planning, antenatal more than 4 visits DPT 3 coverage, child care seeking suspected pneumonia, tuberculosis effective treatment HIV antiretroviral treatment, insecticide treated bed nets, at least basic sanitation, non-raised blood pressure, mean fasting blood glucose, cervical cancer screening, non-use of tobacco, hospital based density, health worker density, access to essential medicine, IHR core capacity index. It is constructed, first within each of the four categories (Reproductive maternal new born and child, infectious disease control, non-communicable disease, service capacity and access), and then across the four category-specific means to obtain the final summary index (39). Geometric means were used instead of arithmetic means as they favored equal coverage levels across services as opposed to higher coverage for some services at the expense of others. The mean values of the 16 indicators were calculated from 2000 to 2015.
6. Population below poverty line 2018: National estimates of the percentage of the population falling below the poverty line are based on surveys of sub-groups, with the results weighted by the number of people in each group (40). Definitions of poverty vary considerably among nations. For example, rich nations generally employ more generous standards of poverty than poor nations. It has been taken from Index Mundi is a data portal that gathers facts and statistics from multiple sources and turns them into easy to use visuals. The average for 2000–2015 of each country was used.
7. Socio demographic index (SDI): The Socio-demographic Index (SDI) is a composite indicator of development status strongly correlated with health outcomes. It is the geometric mean of 0–1 indices of total fertility rate under the age of 25 (TFU25), mean education for those ages 15 and older (EDU15+), and lag distributed income (LDI) per capita (41). As a composite, a location with an SDI of 0 would have a theoretical minimum level of development relevant to health, while a location with an SDI of 1 would have a theoretical maximum level.

### Data Analysis

Different sources of data as described above were primarily exported to MS Excel for cross checking and the analysis was performed with SPSS for Window (version 20) (42). As like Hastie et al., all procedure like data export to prediction was carefully done (43).

1. Data was verified one more time and to assure that there were no errors.
2. Data were analyzed with univariate, bivariate, and multivariate model.
3. Mean and standard deviation were calculated of above dependent and independent variables.
4. Pearson correlation was used as bivariate analysis.
5. Finally, stepwise hierarchical regression model was used to observe strength of association among dependent and independent variables (44).

## Results

For this research, results from 118 countries were used and the mean of the above seven variables of UMIC, LMIC, and LIC were presented. Government health expenditure is 6% in UMIC and about 5% in LMIC and LIC, population below poverty line is more than 2-folds higher in LIC in comparison UMIC, UHC index, and SDI indexes are similar in LMIC and LIC and slightly higher in UMIC (Table 1).

**Table 1:** Mean and standard deviation of used variables in UMIC, LMIC, and LIC.

**Index/indicators/variables**	**Categories of countries (Mean** **±SD)**			
	**UMIC**	**LMIC**	**LIC**	**Average LMIC**
Government Health expenditure in %	6.53 ± 1.87	5.11 ± 1.56	5.64 ± 1.65	5.78 ± 1.74
Population below poverty line in %	15.9 ± 9.9	26.93 ± 14.21	36.68 ± 19.27	25.06 ± 15.74
UHC index	70.75 ± 4.52	57.62 ± 9.19	39.40 ± 4.27	58.29 ± 13.85
Governance index	5.28 ± 1.21	5.24 ± 0.79	4.42 ± 0.61	5.01 ± 1.00
Stability index	−0.34 ± 0.73	−0.57 ± 0.91	−1.50 ± 0.92	−0.71 ± 0.93
SDI index	0.70 ± 0.02	0.58 ± 0.08	0.39 ± 0.08	0.58 ± 0.13
GNI per capita in $	19743.75 ± 6665.68	8023.75 ± 2684.29	3184.00 ± 1517.90	11336.19 ± 8234.80

*UMIC, upper middle-income countries; LMIC, lower middle-income countries; LIC, low income countries; UHC, universal health coverage; SDI, sociodemographic index; GNI, gross national income; SD, standard deviation*.

Table 2 shows the correlation between each variable. There is a positive association between government health expenditure, governance index, stability index, SDI index, and GNI per capita and negative association for population below poverty and the UHC index. There is high correlation between UHC index with governance, stability, and SDI index.

**Table 2:** Raw correlation analysis between independent variables and UHC index for low- and middle-income countries.

**Variables**	**Government Health expenditure in %**	**Population below poverty line in %**	**UHC index**	**Governance index**	**Stability index**	**SDI index**	**GNI per capita in $**
Government Health expenditure in %	1						
Population below poverty line in %	0.416^*^	1					
UHC index	0.309	−0.537^*^	1				
Governance index	0.114	−0.202	0.712	1			
Stability index	0.351	−0.447^*^	0.776^**^	0.732^**^	1		
SDI index	0.147	−0.480^*^	0.799^**^	0.416	0.274	1	
GNI per capita in $	0.052	−0.419^*^	0.671^**^	0.162	0.406^*^	0.712^**^	1

* *Correlation is significant at the 0.05 level (2-tailed)*.

** *Correlation is significant at the 0.01 level (2-tailed)*.

*UHC, universal health coverage; SDI, sociodemographic index; GNI, gross national income*.

### Regression Analysis

Multivariate analysis was performed using a hierarchical linear regression model. The data were interpreted by standard beta coefficients and *p*-values at the significance level of <0.05. According to our full regression model, governance, stability, and SDI index were associated with significantly increased UHC index by 0.33, 0.41, and 0.57 (*p* < 0.05) in model 6 (Table 3). The R^2^ and adjusted R^2^ increasing in each model and all variables explained properly.

**Table 3:** Regression analysis between independent and UHC index for low- and middle-income countries.

**Independent variables**	**Dependent variable (UHC index)**											
	**Model 1**		**Model 2**		**Model 3**		**Model 4**		**Model 5**		**Model 6**	
	**Beta coff**.	***p*-value**	**Beta coff**.	***p*-value**	**Beta coff**.	***p*-value**	**Beta coff**.	***p*-value**	**Beta coff**.	***p*-value**	**Beta coff**.	***p*-value**
Government Health expenditure in %	0.30	0.063	0.202	0.320	0.183	0.358	0.176	0.245	0.165	0.098	0.152	0.065
Population below poverty line in %			−0.493	0.023	−0.243	0.037	−0.173	0.308	−0.138	0.706	−0.135	0.733
Governance index					0.442	0.021	0.390	0.036	0.335	0.047	0.330	0.047
Stability index							0.769	0.002	0.427	0.008	0.412	0.017
SDI index									0.556	0.004	0.575	0.011
GNI per capita in $											0.110	0.486
R^2^	0.09		0.32		0.39		0.67		0.90		0.90	
Adj R^2^	0.04		0.25		0.29		0.59		0.85		0.86	

*UHC, universal health coverage; SDI, sociodemographic index; GNI, gross national income; coff., coefficient*.

## Discussion

This is a cross-sectional study involving of 118 countries in some structural variables. The structural variables of interest for this study were political stability, economy, demographic, governance, and government direct investment in health. We found that there were significant associations between political stability, governance status, and sociodemographic status with universal health service coverage.

Politics, demography, and economy are the major structural factors that need to be developed for each country and obviously also for the universal health coverage too. Our study results highlight the fact that if these factors are not developed at the same pace; UHC will not be successful and cannot be sustained. In our analysis, sociodemographics are strongly associated with universal health service coverage. There are some studies in line with our findings. A study in Nambia concluded that sociodemographic factors (sex, education, and wealth) are associated with universal health care and financial coverage (45). Another study in Ghana also concluded that sociodemographic conditions (elders, women, poor education, and marginalized population) were associated with health services and financial coverage (46). Similar types of study in Vietnam showed that sociodemographic factors influenced health care service and patients satisfaction (47). Those studies conclusions are similar to our findings.

Similarly, political stability is an important factor for the development of a country, particularly in social security. Universal health coverage is one of the major factors in the progress toward social security. A study by Fox et al. reveled that without political negotiation and conflict settlement, health service coverage is hardly possible (48). Bump 2010 concludes that UHC is “intensely political” because it needs construction of consistent programs and policies to provide quality health service to the whole population who bear financial responsibility (49). A model presented by Kelsall et al. showed that political stability creates healthy public policy, adequate funding and improve governance and the achievement of the UHC in a faster way (50). A multicounty analysis by Ranabhat et al. indicated that political instability is negatively associated with universal health coverage and positively associated with immature mortality (51, 52) and there was a high prevalence of tobacco use among youth where health service coverage is poor (53). All the above research findings are in line with our finding.

The governance factor is important in the delivery of health services to the people. The efficiency, transparency, and quick delivery of health care are major concern with governance. In our study, governance is positively associated with universal health coverage. An action plan by World Health Organization concluded that good governance is a prerequisite for UHC (3). An analytical research paper by Fryatt et al. concluded that good governance would support the achievement of UHC and people are accountable within the health system (54). A study from China showed that strengthening health governance significantly improved the health service and health insurance coverage (55). A study by Yeoh et al. on the Asia-Pacific region states that fair governance functions to accelerate progress toward UHC (56). A policy research working paper by the World Bank Human Development Network concluded that governance (fair implementation of health program and policy) accelerates health outcomes and increases the health service coverage (57). The results of those strong studies are similar to our findings.

This study consists of structural factors influencing universal health coverage in low- and middle-income countries. It clearly indicates that increasing government health expenditures, volunteer type of health insurance, poverty alleviation, community empowerment, and even increase of GNI is not sufficient to achieve UHC. More precisely, the results of this study suggest that political stability with a clear policy and commitment, transparency, efficiency, a health system which accountable and equitable society would achieve UHC that could be sustainable. Absolute economic growth is not sufficient to sustain UHC because low- and middle-income countries like Bangladesh, Nepal, India, Cambodia, Libya, and others that have a 6% or higher increase per year but the UHC index is still <50. Thailand, Hong Kong, Turkey, and Malaysia have better UHC indexes and economic growth rate has been stable. More importantly, those countries who already have achieved UHC have sustainable economic growth. So, from application point of view, our study is equally useful for visionary policy makers, academic and professional researchers and expert in this area. It could be more applicable for political masters, bureaucratic leaders, and planners of social security who could be player to settle structural factors of any country. Moreover, we have not highlighted the strength of association (beta coefficient and *p*-value) because adding other variables may fluctuate the results. Important aspect of this finding is such structural factors have high gravity for universal health coverage. In spite of those strengths, this study has some limitations. We are not able to include many other variable related to universal health coverage and statistically, the number of countries involve in this study may not be sufficient and it needs more precautions for generalization of this research outcome. So, more research on structural factors responsible for UHC is strongly recommended.

## Conclusion

To easily achieve UHC, good governance, political stability, and demographic balance are prerequisites and those factors would help any country to achieve UHC by 2030. UHC is a goal and it must be owned by power holders like politicians, policy designers, and program implementers with their different roles and responsibilities. Researcher and expert in this area should coordinate efforts to achieve UHC as the most important aspect of sustainable development goals.

## Data Availability Statement

The datasets generated for this study are available on request to the corresponding author.

## Author Contributions

CR formulated the concept, collected data, prepared, reviewed, and finalized the manuscript. MJ, MD, and C-BK reviewed the manuscript and provided feedback.

### Conflict of Interest

The authors declare that the research was conducted in the absence of any commercial or financial relationships that could be construed as a potential conflict of interest.
